# Development of the PRE-HIT instrument: patient readiness to engage in health information technology

**DOI:** 10.1186/1471-2296-15-18

**Published:** 2014-01-28

**Authors:** Richelle J Koopman, Gregory F Petroski, Shannon M Canfield, Julie A Stuppy, David R Mehr

**Affiliations:** 1 Department of Family and Community Medicine, University of Missouri, MA306 Medical Sciences Building, DC032.00, Columbia, Missouri 65212, USA; 2 Office of Medical Research, University of Missouri, Columbia, Missouri, USA; 3 Center for Health Policy, University of Missouri, Columbia, Missouri, USA; 4 Department of Child Health, University of Missouri, Columbia, Missouri, USA

**Keywords:** Quantitative methods, Measurement issues/instrument development, Information management/informatics, Chronic care

## Abstract

**Background:**

Technology-based aids for lifestyle change are becoming more prevalent for chronic conditions. Important “digital divides” remain, as well as concerns about privacy, data security, and lack of motivation. Researchers need a way to characterize participants’ readiness to use health technologies. To address this need, we created an instrument to measure patient readiness to engage with health technologies among adult patients with chronic conditions.

**Methods:**

Initial focus groups to determine domains, followed by item development and refinement, and exploratory factor analysis to determine final items and factor structure. The development sample included 200 patients with chronic conditions from 6 family medicine clinics. From 98 potential items, 53 best candidate items were examined using exploratory factor analysis. Pearson’s Correlation for Test/Retest reliability at 3 months.

**Results:**

The final instrument had 28 items that sorted into 8 factors with associated Cronbach’s alpha: 1) Health Information Need (0.84), 2) Computer/Internet Experience (0.87), 3) Computer Anxiety (0.82), 4) Preferred Mode of Interaction (0.73), 5) Relationship with Doctor (0.65), 6) Cell Phone Expertise (0.75), 7) Internet Privacy (0.71), and 8) No News is Good News (0.57). Test-retest reliability for the 8 subscales ranged from (0.60 to 0.85).

**Conclusion:**

The Patient Readiness to Engage in Health Internet Technology (PRE-HIT) instrument has good psychometric properties and will be an aid to researchers investigating technology-based health interventions. Future work will examine predictive validity.

## Background

Consumers are turning more to the internet for health information [1]. Online and mobile health interventions to aid lifestyle change and chronic condition self-management are proliferating [2-4]. However, important “digital divides” such as age, education, and rural residence still exist and may limit consumer use of these tools [5]. Concerns about data security, privacy, and lack of motivation may also limit use [6]. Researchers and developers of online and mobile tools may want to assess not only the skills of prospective target patient populations, but also their motivations and concerns, which can be encapsulated in the term “readiness”. Researchers would benefit from an instrument that characterized their research participants’ likelihood of using these technology applications.

There has been previous instrument development in this area. Norman and Skinner developed a 10-item scale, the eHealth Literacy Scale (eHEALS), to measure the eHealth literacy concept. The scale prompts participants to evaluate their own abilities to search for, use, and evaluate health resources on the internet [7,8]. Although this was an important first attempt to measure the concept of eHealth Literacy, it has several limitations. First, the researchers developed the scale with a youthful sample ranging in age from 13 to 21 years. No data exist on its performance in older adults, which is an important limitation, considering that internet and computer skills will likely differ between these two populations. Additionally, a Dutch version of the eHEALS failed to predict internet health use [9]. Lastly, an instrument that goes beyond literacy to measure readiness may be more useful to researchers.

Self-management is an important component of chronic disease management and it is thought that interactive online interventions might engage and support patients to better self-manage [10]. But these tools can only help if patients are ready to use them. Therefore, we developed an instrument designed to go beyond basic eHealth Literacy and computer skills to measure a readiness to use internet resources to access health information. Unlike the eHEALS, we included concepts such as information needs, motivations, privacy concerns, and preferred source of information. Also, we particularly focused on patients with chronic conditions as they tend to be older, a factor associated with decreased internet use [5]. Focusing on those with chronic conditions is important because many of the health information technology interventions are being developed for people with chronic conditions.

## Methods

Table 1 provides an overview of the multiple and iterative methods used in our instrument development process, and the sample size used for each step.

**Table 1:** Instrument development activities

**Activity**	**Participants**	**Items**
4 Focus groups to identify domains	16	---
Literature review of existing scales	---	---
Initial item writing based on domains	---	98
Choosing best items based on best practices	---	53
Feasibility testing/cognitive interviewing	21	53
Instrument development sample	200	53
Test-retest reliability	45	53
Final instrument	---	28

### Identifying domains

To create candidate items, we first sought to understand to the relevant domains. To do this, we conducted four focus groups with 16 patients with the chronic conditions of diabetes, hypertension, heart failure, or coronary artery disease. Separate focus groups with 2–6 participants each were run for self-identified internet users and non-users as it was hypothesized that their issues with technology use would be different. Focus group participants were asked where they got information about their health, internet use, concerns about using the internet for health, information and communication preferences, and past experiences. Focus groups were audio recorded and transcribed by an experienced qualitative transcriptionist. Transcripts were analyzed using grounded theory methodology with investigator consensus on codes and themes, assisted by NVivo 8.0 software. Investigators were RJK a family physician clinical researcher, SMC an MPH experienced in qualitative methods, and JAS a medical student trained by the team in focus group and qualitative methods. All three participated in focus group facilitation and field note recording. RJK and SMC conducted analysis of the focus group data. Both independently coded the transcripts, and then met to agree on codes. Major themes emerged, which then informed the item-writing to capture that domain. Some items were near direct quotes from participants.

### Creating candidate items

Once relevant domains were identified from the focus group themes, we searched the literature for instruments addressing these domains. We identified instruments that addressed internet use [11], internet use for health purposes [12,13], computer and internet anxiety [14], computer and internet abilities [15-18], attitudes and beliefs about the internet [14,19-21], risk perception [22-28], internet security and privacy [6], health literacy [7,29-32], motivation [33,34], and media literacy [7]. While items were not culled from these instruments, reviewing them allowed us to examine different approaches to relevant domains and aided in our overall task of item writing. Item writing was also informed by the focus group themes, including some items that were near direct quotes of participants. Items were written only in the English language.

Of 98 candidate items, we selected the 53 best items, eliminating those that were double-barreled, lengthy, or had large words or complex sentence structure, while maintaining coverage of our identified domains. We avoided jargon, value-laden words, negatively worded questions and negative prefixes, all of which can decrease an item’s validity coefficient [35,36]. We also ensured that every question could be meaningfully answered by both internet users and non-users. Questions are also not specific for any disease and more generally reference “health”. These 53 items made up our candidate item questionnaire. We used a 4-point Likert scale for all items with anchors Strongly Disagree, Disagree, Agree, and Strongly Agree [37]. Items which conceptually might be negatively associated with health information technology use were mixed in with positively associated items to decrease rote responding; these were then scored in reverse order [38].

To ensure that respondents interpreted questions as intended, we administered the 53-item questionnaire to 21 participants in person. We used cognitive interviewing during questionnaire completion, asking them to verbalize their interpretation of the questions as well as the thoughts that led to their answers [35]. The questionnaire was iteratively refined until participants no longer expressed any ambiguity about the meaning of items. During this process a 4-point Likert scale with questions framed as hypothetical scenarios (e.g. If I went on the internet, I would find it frustrating.) was preferable to a 5-point Likert that included a neutral response option. The cognitive interviewing process revealed that participants used the neutral option as a catch-all. The 53-item questionnaire was then administered to 200 additional participants. Information about age, general health status (self-rated), highest level of education, and race/ethnicity was also collected from each participant. Five questionnaires that had more than five skipped questions were omitted for a final development set sample size of 195.

### Sample

All participants for each phase of the instrument development were patients age 18 years and older with the chronic conditions of diabetes, hypertension, heart disease, or heart failure. Patients were all ambulatory patients attending one of 6 family medicine clinics of the Department of Family and Community Medicine at the University of Missouri. Patients were recruited from the waiting room of the clinic, and were asked to answer a screening questionnaire that ascertained if they had a chronic condition. The researcher made efforts to approach every person in the waiting room and made no assumptions about eligibility or experience using internet/computers. We limited participants to those who primarily speak English. The same recruitment method was used for both the focus groups and questionnaire completion. The University of Missouri Health Sciences Institutional Review Board approved all phases of this study.

Given the multiplicity of analyses employed in the development of a new instrument it is difficult to derive a priori sample size estimates. We approached the sample size issue by estimating the sample required for an exploratory factor analysis (EFA). As a starting point, we posited that the propensity to use health information technology is composed of the five factors Capability, Access, Motivation/Risk Perception, Information Needs, and Privacy/Trust. We further conjectured an initial screening step would reduce the number of candidate items to between 40 and 50. We also made the worst-case assumption that items comprising these five factors would exhibit weak communalities (percent of variance in the observed items explained by the factor model). Under these assumptions, guidelines indicate that a minimum sample size of 130 subjects would be sufficient for the factor analysis [39]. To allow for possibility of retaining fewer items or deriving more factors the sample size was inflated to 200 subjects.

### Factor analysis

The first quantitative analysis was to examine item response frequencies for the 53 items in the development sample. Items with insufficient variation across response options were considered for deletion. Such items do not discriminate between different levels of the trait they are intended to measure. After item culling we conducted an EFA to determine the factor structure of the instrument. One can use factor analysis in either an exploratory or confirmatory mode, but since this is a new tool we began with exploratory techniques. With EFA we do not specify the number of factors or the items that load on those factors in advance but rather let the data guide us [40].

An essential step in an EFA is to determine the number of factors to retain. Determining the number of factors is both a matter of judgment on the content and quality of the factors, and a statistical issue. By convention one retains factors with an eigen value of greater than 1.0. This criterion is motivated by the fact that EFA operates on the item correlation matrix wherein item variances are fixed at 1.0, and so factors with a variance (eigen value) less than that of a single item are essentially comprised of noise. The initial factoring extracted 13 factors, however several of the factors were comprised of few items, items that cross-loaded with other factors, or that were difficult to interpret. Final solutions were derived after a Promax oblique rotation. Items with factor loadings of less than 0.30 or with substantial loadings on more than one factor were excluded from the instrument. From the initial pattern matrix of 13 factors, we identified 8 strong factors. We selected groups of items in each factor that loaded most heavily on that factor, and with minimal or no dual loading on other factors. The investigators examined the items in each factor and agreed on a name for the overall factor concept. Each item was assessed and was kept if it had a high loading, was a good conceptual representation of the named dimension, and was well worded and clearly measured the variable of interest. The EFA was re-run with the candidate items to achieve the final instrument. We examined the internal consistency of each factor using Cronbach’s alpha [35,41].

To investigate the possibility that our 8 identified factors clustered into one or more higher order factors, we examined a scree plot of eigen values of the augmented correlation matrix against the number of factors. Factors with eigen values > 1 are candidates for higher order factors. Potential higher order factor solutions were examined using exploratory methods to examine whether the eight factors represent different constructs or whether they reflect different facets of multiple higher order concepts. The preferred way to do this is using confirmatory factor analysis (CFA) techniques, however this analysis had convergence issues, possibly reflecting that the sample size may not be sufficient to support a CFA model with 28 observed variables in 8 factors. Therefore the less demanding EFA method was again used, this time where the raw data is the summated factor scores.

### Test/retest reliability

Three months after the initial sample, we mailed retest questionnaires (full 53 item questionnaire) to a randomly selected sample of 53 participants. 2 declined. Of the 51 remaining, 45 returned completed questionnaires. The eight factors were compared in the initial and retest using Pearson’s correlations.

## Results

Focus group participants were 12 women and 4 men. Twelve self-identified as computer and internet users and 4 were non-users. Their average age was 56 (range 27–75). The percentage of non-internet users participating in the focus groups was similar to the percentage among patients that were approached to participate (25 vs. 32%). The major themes identified in the focus groups with users and non-users revolved around barriers and facilitators to health information technology resource use. Barriers and facilitators are presented in Table 2, with definitions and supporting quotes. One theme, asynchrony, was viewed by some as a facilitator and by others as a barrier.

**Table 2:** Focus group themes

**Theme**	**Definition**	**Supporting quote**
**Barriers**		
**Privacy and security concerns**	Multiple participants, both users and non-users shared this concern, although experience and expertise with computers seemed to dispel concerns for some users. Participants expressed concern that personal information could be leaked when they accessed a website or entered personal information into a system. In addition to fear of privacy infringements, participants were also concerned that viruses, cookies, phishing and spam might lead to security breaches such as identity theft. Users discussed these possibilities as wary consumers while non-users abstractly referred to stories that they had heard in the media that may or may not be relevant, such as a highly publicized Ponzi scheme scandal.	“Cause like there’s a lot of other people out there in the world that, take for instance if I was getting on the internet and so and so said such, the other person’s name, their last name, social security number, you can get all that stuff down from anybody, and it’s dangerous.”
		“I don’t ever, ever use my identity on there [internet] because there’s a lot of viruses’ going on and people just log right in.”
**Poor computer and search abilities**	Non-users described gaps in knowledge and abilities that limited them from accessing the internet, including poor internet navigational skills, lack of virus protection leading to poor computer function, and inability to set up their computer and/or internet access. Both users and non-users expressed that difficulty with spelling could hamper searches, and that it could be difficult to find the information that they needed online. Many expressed that the amount of information on the internet could be overwhelming.	“Oh, yeah. I had to get over that, and my daughter says it’s just like the keyboard of the typewriter and, you know, you just gotta know how to do it.”
		“You know, yeah, you look it up, you look it up and then you don’t know where to click, you know, and stuff like that. That, then it confuses you and then it just, you know, has all those kind of things, you know, written down and I thought “Oh, my God.” It’s just frustrating.”
**Preference for the health care team as a source of information**	Both users and non-users thought that information from the doctor was superior in validity and quality to most information that could be found by them on the internet. They also valued their relationship with their doctor and felt that the doctor served needs that could not be addressed by the internet. Some internet users still sought health information on the internet but it was not used as a primary source of information and the patient often would consult with the care provider to verify information.	“I want something more than that [internet search]. Maybe I want the hands on, you know, and I don’t get the hands on from a computer. I’m sorry. I just don’t get the same feeling from that.”
		“Yeah. I always have to have that doctor to reassure me. A computer couldn’t take the place of a doctor for me.”
**Anxiety about what information might be found on the internet**	Both user and non-user participants had concerns that information found on the internet might increase their anxiety. For example, many stated and agreed that information about medication side-effects might lead them to imagine that they were having those side-effects. Similarly, looking up information about symptoms might lead them to discover a deadly diagnosis. For internet users there was often a tension between avoiding information which might provoke anxiety while also feeling a need to gather more information.	“…and I said “Once I heard about malignancy and early death, I don’t want to hear any more.” And he said “Well, I didn’t want to say that to you, but I wanted to give you the option to find it.” So we can sometimes not want to look in places that we don’t want to find out certain things.”
		“First time I read it, you know, did it all, like on all three websites just telling me all this stuff that could be wrong with me, you know, I’m like “Oh, my God, I’m gonna die,” you know.”
**Facilitators**		
**Need for information**	Participants described that often their desire to find information was triggered by an event creating a specific information need, which prompted the participant’s utilization of the computer. Participants frequently reported a need to obtain information that could help explain a symptom. Participants also reported wanting to learn ways to deal with health issues utilizing “alternative medicine” methods. The information need provoked anxiety, and if this need could not be conveniently or adequately fulfilled by trusted sources, such as the health care team, friends, or family members, then users turned to the internet. They might also triangulate the validity of information based on multiple sources.	“Well, I fell a couple weeks ago and I really don’t know what happened. We think it was insulin reaction, but I felt bad enough that I went to the hospital. And so I have, you know, kind of looked up, looked that up.”
		“Matter of fact, [Doctor Name], and don’t ask me for [Doctor’s] name, she’s a diabetes doctor, told me about a site to go to for we’ll say home remedies for fungal infections of the feet and that kind of stuff, but basically other people will say “I just read an article on,” and I will say “Where was it?” “Medline?” and then I can go find that article.”
**Desire to be a more active participant in own care**	While not common, a few participants strongly expressed that the knowledge that they gained on the internet was a type a power that they used with the doctor to improve their own care. Some expressed that they felt they needed knowledge to more effectively converse with the doctor or to make the doctor take them more seriously or spend more time with them. Others an active role in their own care coordination and management, updating their care team on changes in their health, and learning about behavioral changes and other interventions for better chronic disease care.	I want to be a little bit smarter, too, and I don’t want to just wait, like Dr. [name] was like “Well, I’m gonna examine you, but is there anything else you’d like to address,” and I was saying nothing before.”
		“If I read something or become aware of something one way or the other, I’ve always checked it out with my primary family practice physician, who has been very good about giving me an opinion as to what I’ve said or, or when I’ve brought up, and that’s worked out real well for me.”
**Convenience**	Whether using the computer to communicate with the health care team or to look up needed information, the computer and internet were felt to be very fast and convenient for established users. Non-users also recognized this convenience, but lamented that it was not available to them.	“Well, I use the internet a lot for information regarding health relations, treatment plans. My favorite sources are hospitals that publish patient teaching education. I try to make sure the site is authoritative and not Wikipedia, those kinds of things. I contact my physician with questions.”
		“As I say, it’s just a world of information out there, and that’s one of the fastest way of getting it now, what used to be telephones, write a letter.”
**Looking for information for or about others**	For most users, looking up health information on the internet was an activity that they did almost as often for family and friends as for themselves. In situations where the user was a caregiver, they might even do more searches for health information for loved ones than they would for themselves. Obtaining information for others seemed to be one way that friends and family members involve themselves in caring for people they care about. Additionally, users may look up health information about the conditions of family and friends because they have a relative lack of information about the family/friend health condition, compared to their own health condition where they would receive information directly from the health care team and have an opportunity to have their question answered by the health care team. Similarly, non-users reported that family and friends would look up information for them.	“I have a friend who has schizophrenia, and when I was first trying to find out more about that ailment. I knew very little. ”
		“…my husband has diabetes and strokes, and he’s wheelchair, and my mother is going through chemo for the second time…if there’s something I’m not familiar with, if there’s something I question about what the doctor’s told me, you know, prescriptions, you know, different drugs and exactly what they are, that sort of thing.”
**Barrier & Facilitator**		
**Asynchrony**	Asynchrony occurs when communications are not occurring directly or in real time. This communication between patient and the care team can take place electronically in the form of emails or secure patient portal communications. Patients had divided views of this asynchrony. Some viewed it as a barrier similar to privacy/security because they were not sure where their message would go, who would see it, and when or if they would get a reply. The opposite view reflected the convenience of this asynchrony, with the doctor/nurse and the patient themselves being able to communicate at a time that was most convenient or comfortable for them.	“I don’t want to add another [task], I look at them sometimes run from room to room, and thinking and do you expect them to custom answer my email? And I know one of the downsides of email is the sender never knows how many the recipient is getting…but each person thinks you should give a response immediately, and it’s not possible.”
		“Because you can do that, you know, a lot of times I’m up quite late, so at 1:00 in the morning…I can do it right then because, you know, a lot of times during the day I, I don’t have time to do this. It would just be able to do it on my time and when I’m, when the house is quiet and I’m able to, to concentrate a little bit more.”

Both users and non-users had privacy and security concerns about sharing information online, however users were more sophisticated in their assessment of these potential risks, while non-users had a more global, unfocused concern, almost paranoia, about these risks, likely representing their more limited understanding of the structure and function of the internet. Anxiety about health concerns seemed to work in two ways for the participants. Searching for information was a way to understand better and perhaps relieve information needs and therefore anxiety. However many participants expressed a “no news is good news” approach, reflecting that seeking information on the internet could lead them to more information than they needed, and that some of that information would be distressing. Both groups also expressed that it was easy to become overwhelmed in the sheer amount of information that could be found on the internet, and for the propensity to go “round and round” in their search for information. Looking for information on behalf of others was definitely a prominent activity, as has been found in other literature [42]. Perhaps one of the most desired aims, several users stated that the internet helped them understand their condition more so that they could ask better questions during their visit and become more active participants in their own care.

Two hundred participants with chronic conditions completed the 53-item questionnaire plus demographic questions. The mean age of the sample was 54 years (s.d. 14 years) with a range of 20–86 years. Other demographic characteristics of the sample are listed in Table 3. We had a 64% participation rate among eligible participants approached; some of the eligible participants felt too ill to participate while in the waiting room of the clinic.

**Table 3:** Demographic characteristics of patient sample for questionnaire (n = 200)

**Characteristic**	**Percent**
Gender male	29
**Race**	
White	79
Black	17
Asian	1
Native American	3
Ethnicity hispanic	2
**Education**	
Less than high school	17
High school graduate/equivalent	25
Trade/Some college	30
College graduate	15
Post graduate	13
**Self-rated health status**	
Excellent	3
Very good	15
Good	37
Fair	38
Poor	7
**Chronic condition** (may have multiple)	
Diabetes mellitus	39
Hypertension	81
Heart failure	11
Coronary artery disease	12

Of the original 53 items, 28 items were retained which sorted into 8 factors in the EFA. The content experts on our team (DRM, RJK, and SMC) were easily able to name the factors based on underlying concepts, supporting construct validity. The items and Cronbach’s alpha for each of the 8 factors are listed below. Test-retest reliability for the 8 subscales ranged from 0.60 to 0.85.

### List of items and Cronbach’s alpha for the 8 factors

Health Information Need - HIN (0.84)

If I went on the internet, I would use it to look up things so that I wouldn’t worry about them anymore.

If I went on the internet, I would use it to look up information about herbals and/or supplements.

If I went on the internet I would use it to look up symptoms.

If I went on the internet I would use it to search for information about my health.

If I went on the internet I would use the internet to find information about medications.

Computer/Internet Experience, Expertise – CIEE (0.87)

If I went on the computer, I would be able to figure out most computer problems that I might run into.

If I went on the computer, I would have access to the internet.

If I went on the internet, I would find using the internet to be easy.

If I went on the internet, I would find using email to be easy.

Computer Anxiety – CA (0.82)

If I went on the computer, I would find using it to be frustrating.

If I went on the internet, I would get frustrated with the amount of information I found about health on the internet.

If I went on the internet, I would find searching for information on the internet would be stressful.

If I went on the internet, I would find sorting through information on the internet to be too time consuming.

Preferred Mode of Interaction – PMI (0.73)

Looking up health concerns on the internet is more convenient for me than contacting a doctor’s office.

I prefer calling my doctor’s office to emailing them.

I email my doctor.

I trust the internet as a source for health information.

Looking up information online about medications is easier than asking my doctor.

Relationship with Doctor – RWD (0.65)

I let my doctor handle the details of my health.

Doctors are my most trusted source of health information.

When I have a health concern, my first step is to contact my doctor’s office.

Cell Phone Expertise – CPE (0.75)

I go online using my cell phone.

I use my cell phone to text people almost every day.

Internet Privacy Concerns – IPC (0.71)

If I went on the internet, I would be very concerned about giving any personal information.

If I went on the internet, I would be concerned it would lead to invasions of my privacy.

No News is Good News - NNGN (0.57)

People today want to know too much about their health.

Regarding my health, I agree with the statement “No news is good news.”

I am concerned about what I might find if I look up health issues on the internet.

**Figure 1:**
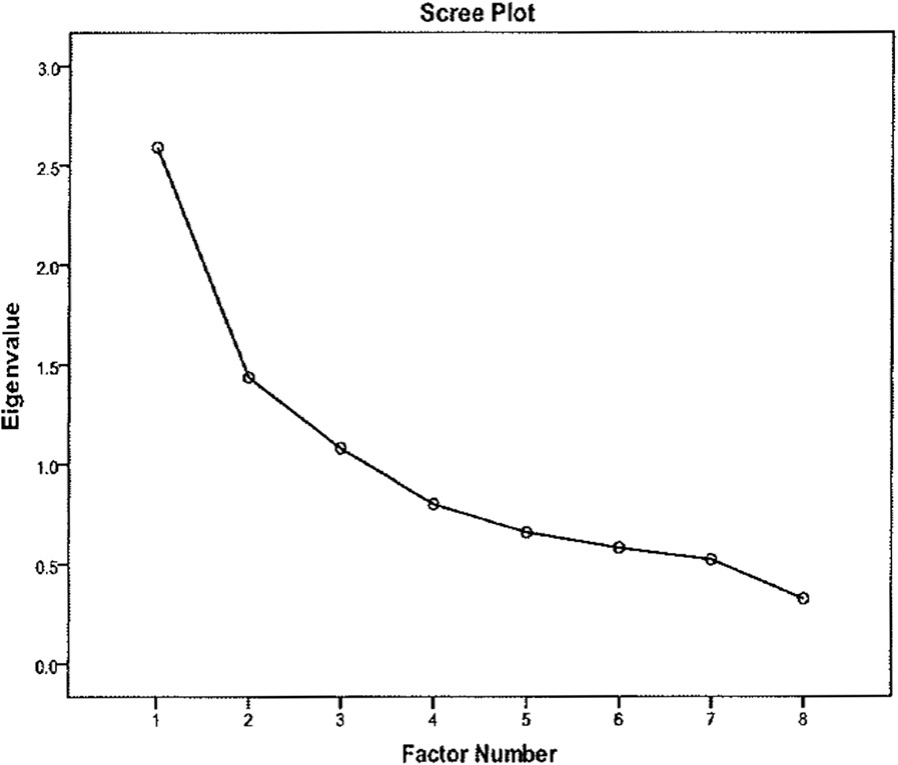
Scree plot of higher order structure.

Examination of a scree plot of eigen values of the augmented correlation matrix against the number of factors revealed the possibility of 2 or 3 higher order factors (Figure 1). Two and three higher order factor solutions were examined using exploratory methods to examine whether the eight factors represent different constructs or whether they reflect different facets of multiple higher order concepts. The three factor solution was problematic with multiple loadings for factors, while the 2 factor solution, Table 4, was both conceptually and analytically unambiguous. Two “meta-factors” were extracted which conceptually represent “Facilitators” and “Barriers” to health information technology use.

**Table 4:** Factor pattern for retaining 2 oblique factors

	**Factor**	
	**1**	**2**
**CIEE**	**0.762**	0.055
**CPE**	**0.614**	-0.286
**PMI**	**0.521**	0.109
**HIN**	**0.486**	0.046
**NNGN**	-0.162	**0.681**
**CA**	0.221	**0.629**
**IPC**	-0.010	**0.516**
**RWD**	-0.039	**0.303**

Factors that clustered with each meta factor are indicated in that column in bold.

## Discussion

The PRE-HIT instrument is a valid instrument to measure likelihood of using health information technology resources among patients with chronic conditions. It addresses using information technology both to search for information and to communicate with the health care team. The instrument demonstrated good test-retest reliability. Its 8 subscales have good construct validity and robust factor loadings. The 8 subscales clustered into 2 larger meta-factors, “Facilitators” and “Barriers”, again, with good construct validity.

There is a good match of the items and factors to the themes identified in the focus groups, reflecting good coverage of the identified domains. Domains and factors include not just measures of computer and internet ability and media literacy that have been addressed by previous instruments, but also user preference for mode of interaction and motivation and desire to search for information.

Women are over-represented in the sample, which likely reflects our strategy of recruiting from our clinic waiting rooms; women make up substantially more than half of all ambulatory care visits, especially as age increases [43]. Women are also the most frequent users of the internet as a health information source, perhaps resulting from their frequent roles as caretakers for children and aging parents [42,44]. The percentages of each race in our sample are similar to the percentages in the United States population, which should aid generalizability [45]. However, Latino ethnicity is under-represented in our sample, so this is also an area for future work.

We limited items to English language and enrolled only participants who spoke English as their primary language. Many of the measure’s domains (e.g. trust, privacy issues) likely have a cultural context far beyond a simple translation and back translation of items. Validating this work for use in other languages and cultures would likely need to examine this cultural context and cultural specificity, in addition to a linguistic translation. Translation and validation in languages other than English is a potential area for future work.

While some factors had a very robust Cronbach’s alpha, others were more toward the low end of acceptable alpha levels. Cronbach’s alpha is very sensitive to the number of items in a factor, and we made the decision to keep the item number small to minimize potential burden on future research participants, perhaps with implications for each factor’s alpha level [41].

The PRE-HIT instrument builds upon groundwork laid by the eHEALS [7]. While the eHEALS was developed in a young population, the PRE-HIT was developed with older adults with chronic conditions. This is a key demographic target of online and mobile health and lifestyle self-management tools. The PRE-HIT instrument also goes beyond the computer skills and media literacy components of the eHEALS to examine factors such as motivation, information needs, privacy concerns, and user preference for mode of interaction [7,11-13,15-18]. The PRE-HIT instrument will likely be better suited to assess readiness among older adults with chronic conditions. This may help to bridge the gap in predicting use that was found with a Dutch examination of the predictive validity of the eHEALS [9].

## Conclusions

Frequently those who are developing and testing new internet and technology based interventions need to enroll patients to test these tools. However, a recurring question is who to enroll, and how to know if the participant is capable of using the technology, and also if they are likely to use it. The PRE-HIT instrument can help researchers choose appropriate test participants. It can also be used to assess a user’s readiness to use the technology and can therefore assist researchers in their statistical analyses evaluating these tools, especially in analyses examining use.

Future work will examine the predictive validity of the instrument, i.e. its ability to predict use of these health technology resources for patients with chronic conditions. This next step will define PRE-HIT scores that are likely to predict use and non-use. Examination of the subscales may also show why a participant uses the technology, or not. Confirming the factor structure and particularly the second order structure is also a target of future research. Additionally, the PRE-HIT instrument is largely suited for addressing computer, internet, and mobile technology use. As technologies evolve, the instrument may need to be modified to address different ways of using technology to improve and inform personal health.

While it would be unrealistic to expect the 28 item PRE-HIT instrument to be used in clinical practice, it will be a great aid to researchers who are examining emerging technologies assist patients with lifestyle change and chronic disease self-management. Currently, it is difficult to determine who to enroll in studies of these technologies and also difficult to characterize the sample beyond simple demographic information. The PRE-HIT instrument will allow investigators to enroll based on specified criteria and to better describe their sample and analyze their results.

## Abbreviations

CFA: Confirmatory factor analysis; EFA: Exploratory factor analysis; eHEALS: eHealth Literacy Scale; PRE-HIT: Patient readiness to engage in health information technology; HIN: Health information need; CIEE: Computer/internet experience, expertise; CA: Computer anxiety; PMI: Preferred mode of interaction; RWD: Relationship with doctor; CPE: Cell phone expertise; IPC: Internet privacy concerns; NNGN: No news is good news.

## Factor names

HIN: Health information need; CIEE: Computer/internet experience, expertise; CA: Computer anxiety; PMI: Preferred mode of interaction; RWD: Relationship with doctor; CPE: Cell phone expertise; IPC: Internet privacy concerns; NNGN: No news is good news.

## Competing interests

The authors declare that they have no competing interests.

## Authors’ contributions

RJK conceived of the study, participated in its design and coordination and drafted the manuscript. GFP participated in the design and performed the statistical analysis. SMC participated in the design of the study, and the analysis and interpretation of data. JAS participated in the analysis and interpretation of qualitative data. DRM participated in the design of the study, and the analysis and interpretation of the data. All authors provided critical review of manuscript drafts, including the final manuscript. All authors read and approved the final manuscript.

## Pre-publication history

The pre-publication history for this paper can be accessed here:

http://www.biomedcentral.com/1471-2296/15/18/prepub
